# Privileged Chiral Photocatalysts

**DOI:** 10.1002/anie.202513320

**Published:** 2025-08-01

**Authors:** Emanuel Studer, Smita Mandal, Timo Stünkel, Ryan Gilmour

**Affiliations:** ^1^ Institute for Organic Chemistry University of Münster Corrensstrasse 36 48149 Münster Germany

**Keywords:** Asymmetric photocatalysis, Light‐enabled reactions, Molecular design, Non‐covalent interactions, Privileged catalysts

## Abstract

Privileged chiral catalysts have transformed asymmetric synthesis, conferring generality to processes that are routinely leveraged in the construction of societally important functional small molecules. Operating in the ground state, these catalysts are conspicuous in their ability to simultaneously regulate reactivity and translate chiral information, often with broad substrate tolerance: this technology continues to expedite chemical space exploration. In stark contrast to the specificity of many enzymatic transformations, this promiscuity affords remarkable latitude for creative endeavour in synthesis. Given the transformative impact that stereoselective photocatalysis has had over the last decade, identifying privileged chiral catalysts that permit reactivity and enantioselectivity to be regulated in excited‐state scenarios has emerged as an attractive but challenging frontier. Providing solutions to address this paradox will require the reactivity/selectivity divide to be reconciled through the validation of chiral scaffolds that effectively operate in non‐ground state environments. Inspired by the venerable treatment by Yoon and Jacobsen entitled “*Privileged chiral catalysts*” (*Science* **2003**, *299*, 1691–1693), this mini‐review is intended to survey the conception and evolution of privileged chiral photocatalyst scaffolds, and offer a perspective on emerging contenders.

## Graphical abstract

Privileged chiral catalysts have transformed asymmetric synthesis, conferring generality to processes that are routinely leveraged in the construction of societally important functional small molecules. This mini‐review is intended to survey the conception and evolution of privileged chiral photocatalyst scaffolds that enable simultaneous orchestration of reactivity and enantioselectivity in non‐ground state regimes.

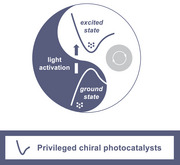

## 1 Introduction

*Sur une Force Jusqu'ici Peu Remarquée qui est Probablement Active Dans la Formation des Composés Organiques (J. J. Berzelius*, ***1835*** *)*.

**Figure 1:**
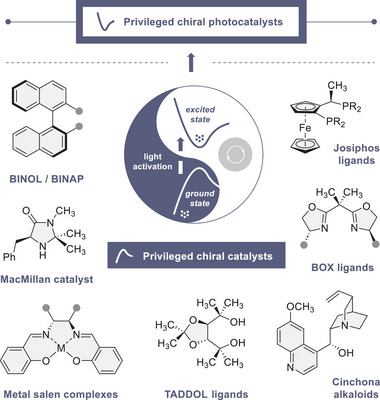
Selected privileged chiral catalysts that operate in the ground state, and the frontier of identifying and validating privileged chiral photocatalysts. Privileged photocatalysts are defined as species that, upon photochemical excitation, simultaneously regulate reactivity and enantioselectivity, and which can engage diverse substrates and display mechanistic versatility.

Selective catalysis is a powerful enabler of precision synthesis and a ubiquitous phenomenon in contemporary chemistry.^[^ ^1^, ^2^, ^3^, ^4^, ^5^ ^]^ Formalized by Berzelius in his foundational 1835 treatise,^[^ ^6^, ^7^ ^]^ the notion of using a small molecule “*interlocutor*” to mediate a chemical reaction has evolved from a curiosity into a major force in shaping academic and industrial research landscapes.^[^ ^8^, ^9^, ^10^, ^11^, ^12^ ^]^ So expansive has been the socio‐economic impact of this discipline, that some 15 Nobel Prizes are associated with the advancement of catalysis science^[^ ^13^ ^]^; many of these recognize key junctures in the development of (stereo)selective transformations.^[^ ^14^, ^15^, ^16^, ^17^ ^]^ In a departure from enzyme specificity,^[^ ^18^ ^]^ small molecule catalysis frequently enables remarkable latitude in stewarding the regio‐ and stereo‐selectivities of molecular processes^[^ ^19^ ^]^: this frequently culminates in generality^[^ ^20^, ^21^, ^22^, ^23^, ^24^ ^]^ that often expedites the construction of societally relevant targets.^[^ ^25^, ^26^, ^27^, ^28^, ^29^ ^]^ This is particularly advantageous in the stereocontrolled construction of chiral small molecules,^[^ ^30^ ^]^ where asymmetric catalysis remains in the vanguard of enabling synthetic methods. Integrating catalytic processes into retrosynthetic logic has eliminated many of the intrinsic limitations associated with chiral pool starting materials,^[^ ^31^ ^]^ diminished reliance on chiral auxiliaries,^[^ ^32^ ^]^ and allowed exciting new avenues such as stereodivergent^[^ ^33^, ^34^ ^]^ and synergistic dual catalysis to flourish.^[^ ^35^ ^]^ In an interesting twist of fate, many of the chiral pool precursors^[^ ^36^ ^]^ conventionally associated with auxiliaries have been repurposed to generate contemporary chiral catalysts; these in turn have the potential to reach broader expanses of chemical space.^[^ ^37^, ^38^ ^]^ This exemplar of evolution in organic synthesis serves to illustrate the ascendancy of asymmetric catalysis as a key technology, and reveals the aptitude of specific molecular architectures to effectively relay stereochemical information. Of the plenum of chiral small molecule catalysts in the contemporary synthesis arsenal, several are considered to be “*privileged*” on account of their ability to orchestrate multiple processes with high levels of efficiency and selectivity (Figure 1).^[^ ^39^, ^40^, ^41^, ^42^ ^]^

Whilst unifying hypotheses that allow structure – generality guidelines to be deduced remain elusive, tangible features are visible in individual molecular architectures. Examples include (*quasi*) *C_2_*‐symmetry (e.g., BINOL/BINAP, salen complexes), the precision orientation of functionality in natural and unnatural scaffolds (e.g., quinine and Josiphos, respectively).^[^ ^43^ ^]^ The bio‐inspired nature of de novo molecular designs is also evident, with the evolution of the semicorrins (e.g. BOX ligands) and their *P*,*N*‐analogs serving as a totemic example.^[^ ^44^, ^45^ ^]^ These observations highlight how chemical intuition has been successfully brought to bear in the creation of a powerful collection of privileged chiral catalysts that operate in the ground state^[^ ^39^ ^]^: moreover, they present an opportunity for creative endeavour in extending the adjective “*privileged*” to chiral catalysts that function in excited‐state regimes.

Energetic considerations are perhaps the most compelling argument for pursuing privileged asymmetric photocatalysts as an emerging pillar in stereoselective synthesis. Processes initiated by photon absorption and excitation, either direct or via sensitization, are not subject to microscopic reversibility.^[^ ^46^ ^]^ It logically follows that harnessing light as a mild, abundant external stimulus to unlock catalysis trajectories that are formally endergonic,^[^ ^47^ ^]^ or thermo‐neutral is potentially expansive. This is exemplified by the rapidly expanding suites of light‐induced *contra*‐thermodynamic isomerization^[^ ^48^, ^49^, ^50^ ^]^ and deracemization reactions,^[^ ^51^, ^52^, ^53^ ^]^ respectively. Capitalizing upon the thermochemical advantages of photo‐activation has thus emerged as a core objective in contemporary catalysis,^[^ ^54^, ^55^, ^56^ ^]^ with chemical intuition taking centre stage in the design and validation of chiral operators. Whilst the notion of coupling asymmetric induction with photochemical activation has important historical foundations,^[^ ^57^, ^58^ ^]^ contemporary molecular designs have unveiled unprecedented generality, thereby expediting molecular assembly.

### 1.1 Enantioselective Photocatalysis

**Figure 2:**
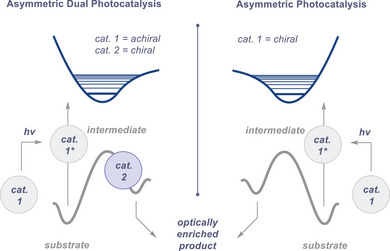
A comparison of dual photocatalysis with asymmetric photocatalysis using a single chiral operator.

A triumph of contemporary organic photochemistry^[^ ^59^, ^60^, ^61^, ^62^, ^63^ ^]^ has been the ability to generate reactive species under mild conditions, thereby enabling their interception in highly productive (stereo)controlled bond forming events. In particular, the last decade has borne witness to the foundation of several conceptual paradigms that enable light‐induced processes to be rendered enantioselective.^[^ ^64^, ^65^, ^66^ ^]^ Whilst a broader discussion is beyond the remit of this mini‐review, it is pertinent to highlight the transformative impact that dual catalysis strategies have had in marshalling light‐generated reactive intermediates towards ground state catalytic cycles in which enantioselectivity is conferred (Figure 2).^[^ ^67^, ^68^, ^69^, ^70^, ^71^ ^]^

The maturation of asymmetric organocatalysis,^[^ ^72^ ^]^ and the expansion of the privileged chiral catalyst arsenal to include secondary amines,^[^ ^41^ ^]^ thioureas^[^ ^19^ ^]^ and Brønsted acids,^[^ ^42^ ^]^ is clearly intertwined with the foundations of asymmetric dual photocatalysis. Expanding the reactivity repertoires of secondary amine‐derived enamines and iminium ions, through the formalization of SOMO activation and its associated reactivity implications, has served as an important bridge to the photochemical strategies that have been explored in the pursuit of enantioselection.^[^ ^73^ ^]^ Exemplars include photosensitization, direct excitation and electron donor‐acceptor (EDA) complex formation.^[^ ^74^, ^75^ ^]^ To these conceptual pillars, strategies that allow selectivity to be directly encoded at the chiral photocatalyst level is a rapidly expanding frontier. This enabling paradigm not only simplifies the challenges associated with multi‐cycle processes, but it also provides an opportunity to democratize excited‐state enantioselective events. Whilst achieving parity with ground state asymmetric catalysis remains a distant goal,^[^ ^20^, ^21^, ^22^, ^23^, ^24^, ^40^, ^41^, ^42^ ^]^ chemical insight and intuition continues to expedite the discovery of chiral photocatalysts that show remarkable versatility across the excited‐state spectrum. This generality, coupled with the ability to explore new reactivity paradigms, will facilitate uptake by practitioners and ensure that this suite of chiral actors expands.

Inspired by Yoon and Jacobsen´s venerable article “*Privileged chiral catalysts”*,^[^ ^39^ ^]^ this mini‐review is intended to survey key milestones in the identification of privileged chiral photocatalysts and offer a perspective on the future of this field of contemporary research.

“Come forth Into the Light of Things, Let Nature be Your Teacher”

William Wordsworth (1770–1850)

## 2 Bach´s Catalyst Design Based on the Kemp Triacid

**Figure 3:**
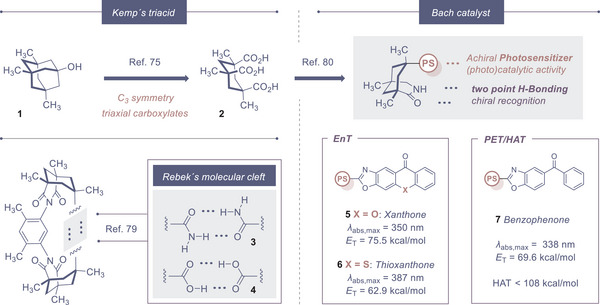
The Kemp triacid (top, left),^[^ ^76^ ^]^ Rebek´s molecular cleft (bottom, left)^[^ ^80^ ^]^ and Bach´s catalyst blueprint^[^ ^81^ ^]^ with selected photophysical data (right).

A survey of chiral photocatalyst design would be incomplete without a laudation for Bach´s iconic blueprint based on the *cis*,*cis*‐1,3,5‐trimethylcyclohexane‐1,3,5‐tricarboxylic acid core; known in common parlance as “*Kemp´s triacid*” (**2**, prepared from **1**. See Figure 3).^[^ ^76^ ^]^ The tri‐axial carboxylate anions of the product provide well‐defined exit vectors for molecular recognition^[^ ^77^, ^78^, ^79^ ^]^ and serve as convenient handles for the generation of a molecular cleft that can be further functionalized: this has been leveraged to great effect by Rebek et al. to create well‐defined microenvironments with directional recognition motifs that emulate biological systems (**3** and **4**, Figure 3).^[^ ^80^ ^]^ In the context of asymmetric photocatalysis, these design principles also manifest themselves in the venerable lactam‐based derivative of Kemp´s triacid developed by Bach and co‐workers.^[^ ^81^ ^]^ In a feat of precision engineering, reciprocal amide‐amide interactions between the chiral host and substrate enable an enzyme‐like templating effect, thereby enforcing a high degree of structural pre‐organization prior to photochemical activation.

Importantly, this modular scaffold has the capacity to accommodate achiral dye motifs proximal to the two‐point binding motif;^[^ ^82^ ^]^ this allows reactivity to be modulated and ensures efficient translation of stereochemical information from the catalyst to the substrate. In the absence of competing background reactions, this approach enables light‐induced processes involving amide‐containing substrates to be rendered enantioselective. This conceptual approach is expansive and has been validated in an array of enantioselective transformations that operate via distinct activation modes (vide infra). Whilst a pre‐condition of this approach is the requirement for an amide binding motif in the substrate, the latitude afforded by this class of catalysts renders it privileged. It is pertinent to note that in the case of triplet energy transfer mediated reactions (EnT), where a pronounced sensitivity to spatial proximity is manifest, subtle structural design aspects can be leveraged to allow selective excitation of one enantiomer: these include the differences in binding constants and distances between the bound substrate and photosensitizer (PS) in the diastereomeric complexes.^[^ ^83^ ^]^ When merged with a subsequent racemization event, this strategy unlocks light‐enabled deracemization in which the inherent decrease in entropy is compensated by photo‐irradiation.^[^ ^84^, ^85^ ^]^ Key to the success of this approach is the condition that catalyst‐substrate binding outcompetes the self‐association of the catalyst.^[^ ^84^ ^]^ A strength of the Bach design is the structural modularity of the catalyst, which is composed of an achiral photosensitizer and a chiral core. Consequently, the photosensitizer can be easily tailored to optimize parameters such as triplet energy and excitation wavelength.

Since exergonic energy transfer is favoured, the triplet energy of the photosensitizer tends to exceed the triplet energy of the substrate: this design approach has proven to be highly effective, resulting in a suite of validated designs. Exemplars include the xanthone (**5**) and thioxanthone (**6**) based sensitizers, which have proven to be highly efficient in energy transfer processes (Figure 3). Xanthone **5** is characterized by a comparatively high triplet energy of *E*_T_  =  75.5 kcal mol^−1[^ ^81^ ^]^ with an absorption maximum (*λ*_abs, max_) at 350 nm. Since direct excitation of the substrate is known to compromise selectivity, leveraging the thioxanthone analog **6** offers a valuable alternative on account of the red‐shifted absorption maximum at 387 nm, and a lower triplet energy of *E*_T_  =  62.9 kcal mol^−1^.^[^ ^81^ ^]^ In contrast, the benzophenone catalyst **7** allows complementary reactivity to be unveiled; this includes photoinduced electron‐transfer (PET)^[^ ^82^ ^]^ and hydrogen atom transfer reactions (HAT).^[^ ^86^, ^87^, ^88^, ^89^ ^]^ This photosensitizer, which is capable of cleaving C─H bonds with bond dissociation energies below 108 kcal mol^−1^^[^ ^90^ ^]^ upon judicious alignment of substrate and sensitizer, has an absorption maximum (*λ*_abs, max_) at 338 nm.

**Figure 4:**
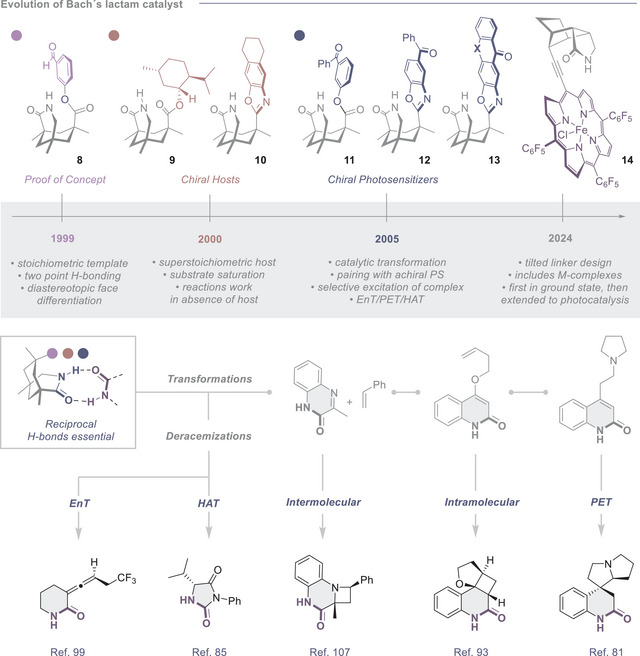
Evolution of the Bach catalyst design and selected applications and activation modes. PS, photosensitizer.

The chronology of Bach´s design begins with a seminal study in 1999 on the application of aldehyde **8** (Figure 4) to enable a highly diastereoselective *Paternò‐Büchi* reaction with a cyclic enamide.^[^ ^91^ ^]^ Upon binding to the racemic linker, the enantiotopic faces of the aldehyde become diastereotopic, thereby enabling facial differentiation in the reaction with the substrate. When deploying optically enriched aldehydes, the observed diastereotopic face differentiation is translated to enantiotopic face differentiation.^[^ ^92^ ^]^ This study constitutes an important proof of concept that the lactam design enables the efficient relay of stereochemical information in photochemical processes. The validation of reciprocal substrate/catalyst binding at two of the three exit vectors of the Kemp acid core then logically led to variation at the site of the third exit vector. Replacement of the benzaldehyde of **8**, which participated in *Paternò‐Büchi* reactions, by sterically demanding menthol (**9**) and tetrahydronaphthalene (**10**) moieties, enabled facial differentiation based on non‐covalent interactions: this culminated in more general chiral complexing agents for photochemical reactions (Figure 4).^[^ ^93^ ^]^ This approach has proven to be expansive, finding widespread application in reactions such as intramolecular^[^ ^94^ ^]^ and intermolecular [2 + 2] cycloadditions,^[^ ^93^ ^]^ *Norrish‐Yang* cyclizations,^[^ ^95^, ^96^ ^]^ 6π‐electrocyclizations^[^ ^97^ ^]^ and *Diels‐Alder* reactions of *o*‐quinodi‐methanes.^[^ ^98^ ^]^

Since the presence of the chiral hosts does not mitigate competitive racemic background reactions, they must be deployed in excess to ensure substrate saturation. Addressing this critical issue led to a major evolution in 2005 when the first catalytic system was reported.^[^ ^82^ ^]^ Replacing the bulky shielding group with an achiral photosensitizer (**11**–**13**) allowed selective substrate‐host excitation to be achieved through selective excitation of the chromophore at longer wavelengths (Figure 4). The report of an energy transfer‐mediated [2 + 2] photocycloaddition in 2009, entitled “*Light‐driven enantioselective organocatalysis*”,^[^ ^84^ ^]^ remains an important benchmark for the field.

The synthetic utility of photocatalysts that are predicated on the combination of precision hydrogen bonding coupled with proximal photosensitizing units is evident in a broad spectrum of transformations, but it is arguably most pronounced in deracemization processes.^[^ ^85^ ^]^ Amide‐based substrates bearing allenes,^[^ ^99^, ^100^ ^]^ sulfoxides,^[^ ^101^ ^]^ cyclopropanes,^[^ ^102^ ^]^ axially chiral alkenes^[^ ^103^ ^]^ and spiro‐cyclopropanes^[^ ^83^ ^]^ are compatible with this catalyst class and allow optical enrichment to be achieved under operationally mild conditions. More recently, this suite of reactions has been extended to include the reversible HAT‐based deracemization of cyclic dipeptides,^[^ ^104^ ^]^ hydantoins,^[^ ^86^ ^]^ oxindoles^[^ ^87^ ^]^ and *N*‐carboxyanhydrides.^[^ ^88^ ^]^ In addition to deracemization, these privileged chiral photocatalysts have taken centre stage in cycloaddition^[^ ^105^, ^106^, ^107^ ^]^ and *aza‐Paternò‐Büchi* reactions.^[^ ^108^ ^]^

Collectively, these studies have galvanized the privileged status of Kemp triacid‐based lactams in contemporary photocatalysis and fuelled interest in the development of structurally related scaffolds that permit reciprocal donor‐acceptor interactions. The tilted tricyclic octahydro‐1*H*‐4,7‐methanoisoindol‐1‐one linker designed by Deslongchamps and co‐workers^[^ ^109^, ^110^ ^]^ has emerged due to its ability to match the greater steric demand of a metal‐bound substrate by increasing the distance between substrate and active site.^[^ ^111^, ^112^ ^]^ Recently, this design was introduced to the field of asymmetric photocatalysis, deploying a chiral iron porphyrin complex **14** for intermolecular amination, utilizing azides as the nitrene source (Figure 4, top right).^[^ ^113^ ^]^ Contrary to many privileged chiral catalysts that operate in the ground state,^[^ ^39^, ^40^ ^]^ the Bach catalyst designs do not harvest the chiral pool.^[^ ^114^ ^]^ The generality associated with these asymmetric photocatalysts derives from chemical intuition and thoughtful design. This enabling technology remains a masterclass in leveraging precision interactions to achieve structural pre‐organization in non‐ground state conditions.

## 3 Octahedral Iridium Complexes in Asymmetric Photocatalysis

Achiral transition metal complexes have a revered history in the advent and development of visible light photoredox catalysis.^[^ ^59^, ^60^, ^61^, ^62^ ^]^ Their success is grounded in considerations of stability, commercial availability and the long‐lived excited states that can be generated by operationally expedient irradiation protocols. Consequently, a plethora of common organometallic complexes has now been repurposed as light‐harvesting antennas that can be predictably deployed to enrich this branch of contemporary synthesis through the mild generation of high energy intermediates.^[^ ^54^, ^115^, ^116^, ^117^ ^]^ In the context of this review of *privileged chiral photocatalysts*, the success of iridium (Section 3) and rhodium (Section 4) complexes bearing *N*‐heterocyclic ligands is particularly conspicuous and inextricably linked to their well‐defined photophysical properties. These octahedral complexes have proven to be particularly effective at regulating single electron transfer (SET) events that would be otherwise challenging in the absence of a photoactivation event. As the field of dual or synergistic asymmetric photocatalysis^[^ ^67^, ^68^, ^69^, ^70^, ^71^ ^]^ is complemented by the growing interest in chiral photocatalysts that govern reactivity and stereoselectivity in non‐ground state environments, this bifurcation has already given rise to effective catalyst designs that may be considered to be privileged. Predicated on the use of chiral transition metal complexes (^“^ *chiral‐at‐metal* ^”^), these approaches share a common theme of leveraging precision (catalyst‐substrate) interactions to ensure structural pre‐organization and subsequently relay stereochemical information. This can be achieved by direct substrate complexation at the metal centre or through interactions with the ligand sphere (vide infra). There is a degree of similarity with the Bach design in so far as the substrates require tailored recognition motifs. Whilst a survey of configurationally stable, chiral‐at‐metal complexes in ground‐state asymmetric catalysis is beyond the scope of this review, and readers are directed to an excellent treatment by Zhang and Meggers,^[^ ^118^ ^]^ it is instructive to highlight selected key milestones to place advances in asymmetric photocatalysis in context. Important foundational studies include those by Fontecave and co‐workers in establishing that octahedral Ru(II) complexes bearing achiral ligands can be utilized in the enantioselective oxidation of sulfides to sulfoxides by hydrogen peroxide.^[^ ^119^ ^]^ Expanding on this report, the same group further validated a chiral‐at‐metal ruthenium complex in the asymmetric transfer hydrogenation of ketones.^[^ ^120^ ^]^ This discussion would not be complete without a mention of Werner complexes in asymmetric catalysis.^[^ ^121^ ^]^ In 2008, Ganzmann and Gladyz reported that enantiopure Werner cations of Co(III) enable the moderately enantioselective *Michael addition* reaction of malonates to enones.^[^ ^122^, ^123^ ^]^ Collectively these examples served to underscore the value of asymmetric reaction blueprints that leverage the chiral‐at‐metal principle in scenarios in which configurational stability is not compromised.

**Figure 5:**
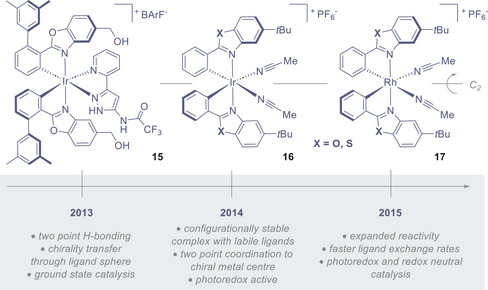
The design of chiral‐at‐metal catalysts by Meggers and co‐workers.

In a major shift towards synthetic utility, two key studies appeared in 2013: the first was the enantioselective ring‐opening/cross metathesis sequence enabled by a resolved, stereogenic at ruthenium complex by Hartung and Grubbs,^[^ ^124^ ^]^ and the second was the use of a chiral‐at‐metal Ir(III) catalyst for enantioselective transfer hydrogenation by Meggers and colleagues.^[^ ^125^ ^]^ Predicated on the notion that the high configurational stability of iridium complexes might allow for ligand exchange whilst retaining the stereochemical information at the metal centre, the latter example would become the first step in a process of evolution that ultimately culminated in a privileged class of chiral photocatalysts (Figure 5). Iterations in the design of a chiral‐at‐metal iridium(III) complex for the asymmetric transfer hydrogenation of β,β′‐disubstituted nitroalkenes (**15**) initially focussed on the role of hydrogen‐bonding to achieve structural pre‐organization.^[^ ^125^ ^]^ To complement this approach of incorporating peripheral ligands with tailored donor units to selectively engage the substrate (**15**), Meggers and colleagues hypothesized that an Ir(III) complex bearing two labile ligands might undergo ligand exchange without compromising optical purity. The configurational stability of complex **16** (X = O) was harnessed to achieve an enantioselective *Friedel‐Crafts* addition of indoles to α,β‐unsaturated 2‐acyl indoles.^[^ ^126^, ^127^ ^]^ Importantly, the bidentate coordination of the acyl imidazole to the iridium(III) centre ensured precise substrate binding and efficient chirality transfer.^[^ ^126^ ^]^ This catalyst design was subsequently validated in photocatalysis, specifically in the asymmetric alkylation of 2‐acyl imidazoles.^[^ ^128^ ^]^ Substitution of the oxygen atom in the ligand scaffold by sulfur **16** (X = S) resulted in a notable enhancement in enantioselectivity: this may be a consequence of the longer C─S bond positioning the sterically demanding *tert*‐butyl groups in closer proximity to the substrate binding site. Shortly after this discovery, Meggers and colleagues introduced the rhodium(III) analog of this complex (**17**),^[^ ^129^ ^]^ and once again demonstrated the improved performance of the benzothiazole ligand.^[^ ^130^ ^]^ Generally, the rhodium(III) complexes exhibit ligand exchange rates that are several orders of magnitude faster than those of the iridium congeners: this accelerates what are often rate‐limiting ligand exchange steps in the catalytic cycle and offer valuable latitude in reaction development^[^ ^130^ ^]^ (vide infra, Section 4).

### 3.1 The Meggers Iridium Catalyst Design

**Figure 6:**
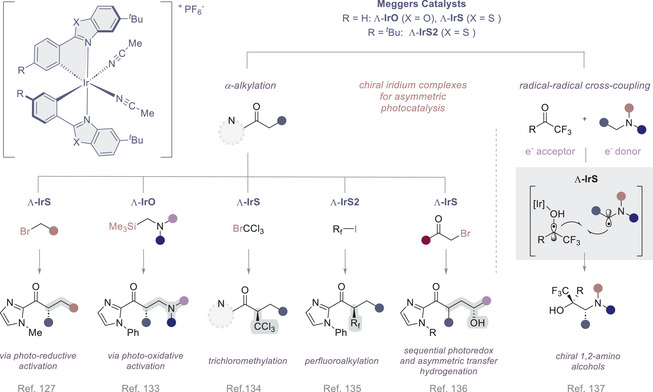
Chiral iridium(III) catalysts developed by Meggers and co‐workers, and the asymmetric transformations that they enable.

The validation of *bis*‐cyclometalated, propeller‐shaped chiral‐at‐metal iridium(III) and rhodium(III) complexes by Meggers and co‐workers continues to have a major impact in shaping asymmetric photoredox cataysis.^[^ ^131^ ^]^ As illustrated in Figure 5, these highly modular, *C_2_*‐symmetric complexes are composed of two cyclometalating ligands, two hemilabile acetonitrile ligands and a hexafluorophosphate counterion. It is interesting to note that the strong σ‐donating ability of the phenyl ligands provides configurational stability to the *bis*‐cyclometalated unit, while simultaneously rendering the acetonitrile ligands labile by virtue of the *trans*‐effect.^[^ ^132^ ^]^ Collectively, these characteristic features work in concert to ensure effective substrate coordination and subsequent asymmetric induction. The favourable photophysical properties of *bis*‐cyclometalated iridium complexes^[^ ^133^ ^]^ were expertly marshalled by Meggers and co‐workers in the chiral iridium complex **Λ‐IrS**, which is an excellent catalyst for the enantioselective alkylation of 2‐acyl imidazoles with electron deficient benzyl bromides and phenacyl bromides (Figure 6).^[^ ^128^ ^]^ In this particular transformation, in which the photocatalyst engages the substrate through Lewis acid activation, photoredox catalysis occurs in a highly stereocontrolled manner to afford α‐alkylated products with high levels of enantioselectivity. A similar substrate‐catalyst activation mode was used to enable the oxidative coupling of 2‐acyl‐1‐phenylimidazoles with *N,N*‐diaryl‐*N*‐(trimethylsilyl)methylamines, albeit via a mechanistically distinct pathway.^[^ ^134^ ^]^ This visible‐light‐activated photoredox catalysis paradigm was further extended to the enantioselective α‐trichloromethylation of 2‐acyl imidazoles and 2‐acylpyridines.^[^ ^135^ ^]^ It is interesting to note that the chiral iridium complex **Λ‐IrS** functions as both a Lewis acid and a competent redox active photocatalyst, and that the process occurs via an electron‐transfer‐mediated nucleophilic substitution via S_RN_1. The authors have further demonstrated the utility of this platform by extending it to enantioselective radical perfluoroalkylation using perfluoroalkyl iodides in presence of **Λ‐IrS2**.^[^ ^136^ ^]^ Merging the photoredox event with a second transformation is potentially expansive and this is exemplified by a sequential photoredox/asymmetric hydrogenation, again using the single chiral‐at‐metal complex **Λ‐IrS**.^[^ ^137^ ^]^ By modifying the catalyst‐substrate activation mode, this same versatile chiral iridium complex (**Λ‐IrS**) facilitated radical‐radical hetero‐couplings: this enabled the enantio‐ and diastereoselective syntheses of 1,2‐amino alcohols.^[^ ^138^ ^]^ The mechanism of the reaction involves photoinduced electron transfer from a tertiary aryl amine to the catalyst‐activated ketone substrate.

### 3.2 The Yoon Iridium Catalyst Design

**Figure 7:**
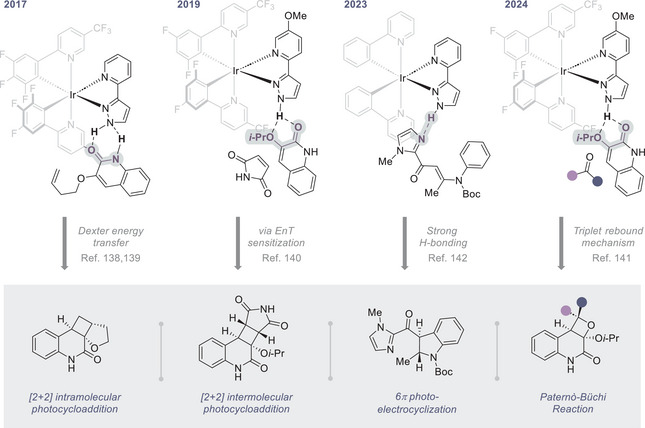
Chiral iridium catalysts developed by Yoon and co‐workers, and the key non‐covalent interactions that are crucial to ensure enantioselectivity.

The success of octahedral Ir(III) complexes in asymmetric photocatalysis originates from the conformational rigidity of the scaffold, the stereochemical information encoded at the central metal atom, and the well‐defined photophysical behaviour upon irradiation. Marshalling these attributes has been key to the design of several highly active catalysts reported by Yoon and co‐workers. In contrast to the strategy outlined in **Section 3.1**, the Yoon design harnesses a peripheral ligand with an integrated docking site that enables reciprocal hydrogen bonding interactions to occur. The success of the design is evident from the range of enantioselective processes that can be achieved with Ir(III) complexes; these include intra‐ and inter‐molecular [2 + 2] cycloaddition^[^ ^139^, ^140^, ^141^, ^142^ ^]^ and 6π‐electrocyclization^[^ ^143^ ^]^ reactions (Figure 7). A key contributor to the success of transition‐metal photocatalysts is their remarkable tolerance towards ligand modification in the pursuit of precision directing units. A compelling example is the report of a chiral Ir(III) triplet sensitizer with a pyridylpyrazole unit that enables hydrogen‐bonding and π‐π interactions: these key interactions orient the substrate to facilitate Dexter energy transfer and ensure high levels of enantioinduction.^[^ ^139^, ^140^ ^]^ A key design consideration is that the acidic N─H bond of the pyrazole interacts with the Lewis basic quinolone carbonyl, thereby ensuring that the intramolecular [2 + 2] cycloaddition occurs in proximity to the chiral transition‐metal photosensitizer. Building on these design principles, the more challenging intermolecular version of the enantioselective [2 + 2] cycloaddition of 3‐alkoxyquinolones with electron‐deficient alkenes was achieved by the same group.^[^ ^141^ ^]^ Thoughtful mechanistic analyses have allowed Yoon and colleagues to delineate the mechanistic differences that distinguish these closely similar reactions. In the latter case, the maleimide substrate interacts directly with the cyclometalating ligand to enable Dexter energy transfer. It is likely that the sensitized maleimide then reacts with the hydrogen‐bonded quinolone‐photocatalyst complex to afford a highly enantioenriched cycloadduct. In expanding the repertoire of their chiral Ir(III) complexes, the authors have effectively utilized them as photosensitizers for highly enantioselective 6π‐photocyclization events.^[^ ^143^ ^]^ In this particular transformation, success is contingent on a key hydrogen‐bond between the pyrazole moiety of the chiral photocatalyst and the imidazolyl ketone of the substrate. Mechanistic studies further delineated the crucial role played by steric factors and torquoselectivity^[^ ^144^ ^]^ in effecting the conrotatory 6π‐photoelectrocyclization. More recently, Yoon and colleagues have illustrated the effectiveness of their Ir(III) photosensitizer design in the enantioselective, catalytic *Paternò‐Büchi* reaction.^[^ ^142^ ^]^ In this process, a triplet rebound mechanism is operational in which the photocatalyst initially engages the quinolone substrate. This is quenched by the ketoester substrate, which further reacts with the initial encounter complex to provide the oxetane in optically enriched form. Collectively, the examples shown in Figure 7 illustrate the importance of directional non‐covalent interactions enabled by a simple heterocyclic ligand design. It is extremely tempting to draw biomimetic analogies with enzyme‐cofactor interplay in refining reactivity.

## 4 Octahedral Rhodium Complexes in Asymmetric Photocatalysis

**Figure 8:**
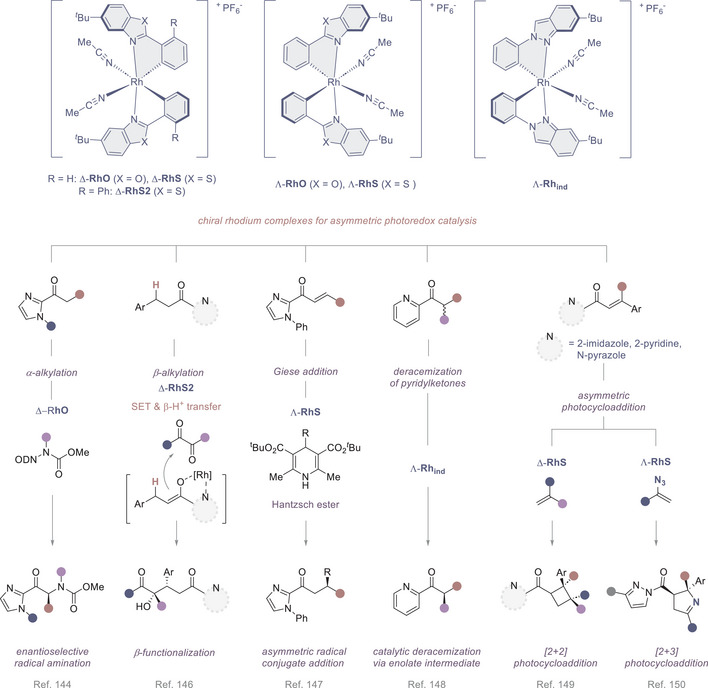
Chiral rhodium(III) complexes developed by Meggers and co‐workers, and their application in enantioselective photocatalysis.

As highlighted in **Section** **3**, the field of asymmetric photocatalysis has been heavily enriched by the introduction of chiral Ir and Rh complexes, and the latitude afforded by the switch in the central metal ion has been thoughtfully described by Meggers and colleagues.^[^ ^129^, ^130^ ^]^ The same group introduced a *bis*‐cyclometalated rhodium(III) complex **Δ‐RhO** as a potent visible‐light‐activated catalyst for the enantioselective radical amination of 2‐acylimidazoles (Figure 8).^[^ ^145^ ^]^ Although, analogous *bis*‐cyclometalated iridium(III) complexes are known to be competent photoredox/chiral Lewis acid catalysts for C─C bond formation,^[^ ^128^ ^]^ these species proved to be ineffective in directing C─N bond formation. This can be rationalized by the rapid ligand exchange permitted in the rhodium complexes, enabling favourable interactions with the transient nitrogen‐centred radicals. The structurally related complex **Λ‐Rh_ind_** is an efficient mediator of visible‐light‐induced asymmetric α‐cyanoalkylation of 2‐acyl imidazoles.^[^ ^146^ ^]^ The authors observed that on exchanging the complex for *bis*‐cyclometalated phenylbenzothiazole **Λ‐IrS**, high levels of enantioselectivity were achieved albeit at the expense of efficiency. The low yield of 23% was attributed to catalyst inhibition, in which coordination of either the bromoacetonitrile substrate or the cyanoalkylated product suppresses activity. This explanation aligns well with the observation that the *bis*‐cyclometalated iridium catalyst exhibits significantly slower ligand exchange kinetics compared to its rhodium analog: this renders it more susceptible to deactivation in the presence of competing coordinating groups.

Structural modification of the core to generate **Δ‐RhS2** opens the possibility to realize the asymmetric β‐C(sp^3^)‐H functionalization of 2‐acyl imidazoles and 2‐acylpyridines with 1,2‐dicarbonyl compounds.^[^ ^147^ ^]^ Visible‐light excitation of the rhodium enolate intermediate triggers a SET to the 1,2‐dicarbonyl compound, which subsequently undergoes proton transfer and stereocontrolled radical–radical recombination.

Using 4‐alkyl‐substituted Hantzsch esters as radical precursors, Meggers and co‐workers have developed an enantioselective β‐functionalization of α,β‐unsaturated 2‐acyl imidazoles using the chiral Lewis acid and photoactivating agent **Λ‐RhS**.^[^ ^148^ ^]^ This approach delivers essentially quantitative yields of the target product with high enantioselectivities, and constitutes a practical and efficient redox‐neutral process driven by electron transfer catalysis. A seminal example of catalytic α‐deracemization of ketones via sequential photoredox deprotonation and enantioselective protonation was reported in 2021 using a **Λ‐Rh_ind_** species.^[^ ^149^ ^]^ While the principle of microscopic reversibility precludes catalytic one‐step deracemization via a deprotonation–reprotonation pathway, Meggers and co‐workers were able to overcome this constraint by engaging photoredox‐driven deprotonation via single‐electron transfer (SET) followed by a HAT step.

Notably, the chiral rhodium complex acts simultaneously as a photoredox/chiral Lewis acid catalyst, whilst the tertiary amine cocatalyst orchestrates three sequential functions: namely as a single‐electron donor, hydrogen atom acceptor, and ultimately as the proton source. In the field of cycloaddition chemistry, the chiral rhodium complex **Δ‐RhS** has been shown to catalyze intermolecular [2 + 2] cycloadditions, affording a broad spectrum of cyclobutanes with excellent levels of enantio‐ and diastereo‐selectivity.^[^ ^150^ ^]^ The propensity of rhodium to engage in azide degradation chemistry provided a foundation for the development of a visible‐light mediated catalytic asymmetric [2 + 3] photocycloaddition of alkenes with vinyl azides.^[^ ^151^ ^]^ In the presence of **Λ‐RhS**, a broad range of 1‐pyrrolines could be accessed as single diastereomers with exquisite levels of enantiomeric excess. The breadth of photocatalytic reactions enabled by chiral‐at‐metal‐Rh(III) complexes has been further expanded to include the asymmetric dearomatization of heteroaromatics using visible‐light‐activated [2 + 2] photocycloadditions.^[^ ^152^ ^]^ Once again, the chiral Lewis acidic photocatalyst **Δ‐RhS** coordinates to the pendant *N*‐acylpyrazole unit of the substrate, thereby enabling the synthesis of chiral tricyclic compounds in a stereocontrolled fashion.

The advances surveyed in Sections 2–4 have revolutionized the field of asymmetric photocatalysis on account of the spectrum of transformations that are enabled under the auspices of three privileged catalyst scaffolds. Through logical processes of molecular design, the power of directional non‐covalent interactions has been exploited in both organic and organometallic designs in non‐ground state environments. The simultaneous orchestration of reactivity and selectivity through effective conformational control enables optical enrichment to be achieved using a single chiral catalyst. An important condition of these approaches is the requirement for tailored directing groups on the substrate and catalyst. Whilst these enabling approaches confer a variety of practical advantages, accessing the catalyst of interest requires investment in multi‐step synthesis campaigns.^[^ ^114^, ^127^, ^131^ ^]^

## 5 Al‐Salen Complexes

Aluminium salen complexes (Al‐salen) are emerging as attractive chiral photocatalysts^[^ ^153^ ^]^ on account of their relative abundance, low cost and well‐defined photophysical properties.^[^ ^154^, ^155^ ^]^ Already considered “*privileged*” in the ground state (see Figure 1),^[^ ^156^, ^157^, ^158^, ^159^ ^]^ these chiral catalysts are straightforward to prepare and their modularity lends itself to fine‐tuning performance. Whilst the stereochemical information is typically encoded by readily available, chiral 1,2‐diamine backbones (*quasi‐C_2_* symmetry), the ligand sidearm and ancillary ligand provide handles to modulate Lewis acidity, redox potential, photophysical properties and conformation. In addition, many Al‐salen complexes are bench stable, which further adds to their appeal.^[^ ^157^ ^]^

**Figure 9:**
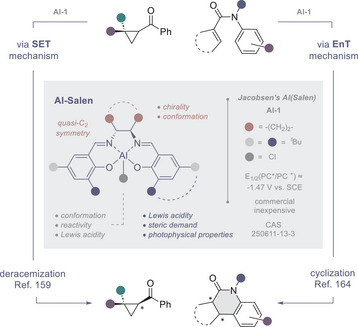
Deracemization and enantioselective cyclization enabled by a commercial Al‐salen complex (**Al‐1**).

This laboratory has demonstrated that a commercial, chiral complex (**Al‐1**) is a highly competent photocatalyst for the deracemization of cyclopropyl ketones (Figure 9).^[^ ^160^ ^]^ Building on a previous interest in cyclopropane activation,^[^ ^161^ ^]^ together with a report by Knowles and Houk on complexation‐induced bond weakening,^[^ ^162^ ^]^ the feasibility of this reaction was explored. Harnessing the reducing power of the ligand chromophore [*E_1/2_* (PC*/PC^+^) −1.47 V vs. saturated calomel electrode (SCE)], it was envisaged that generation of a transient ketyl radical should be feasible by electron transfer from the excited‐state catalyst to the substrate. The intimate involvement of the complex in the reverse process would ensure it occurred in a chiral environment, thereby leading to optical enrichment. Importantly, this single electron‐transfer (SET) process is not reliant on tailored substrate recognition units.

With a view to demonstrating mechanistic diversity in Al‐salen photocatalysis, validation of an energy transfer (EnT) mediated enantioselective photocyclization of acrylanilides has also been investigated.^[^ ^97^, ^163^ ^]^ Utilizing the same commercial catalyst (**Al‐1**) as for the deracemization of cyclopropyl ketones via SET, it was possible to generate a variety of heterocyclic products with high levels of enantioselectivity.^[^ ^164^ ^]^ Collectively, these studies illustrate the potential of ubiquitous Al‐salen complexes in simultaneously regulating reactivity and selectivity in the excited‐state. Their pedigree in asymmetric ground‐state catalysis, ease of preparation and efficiency in the aforementioned processes create a powerful impetus for further investigation.

## 6 Conclusion

Identifying chiral photocatalysts that permit reactivity and enantioselectivity to be controlled in excited‐state scenarios is an emerging frontier. Despite the obvious challenges associated with this enterprise, several scaffolds have emerged that show remarkable generality, stewarding highly enantioselective processes by unlocking discrete activation modes upon operationally simple irradiation. The success of these molecular designs is a triumph of chemical intuition, thoughtful design and *post facto* rationalization, but sustained innovation will be contingent on expanding the current suite of chiral catalysts. This will require a concerted effort to identify candidates that have well‐defined photophysical properties and which can be utilized under operationally simple conditions. Whilst contemporary designs expertly leverage reciprocal donor‐acceptor interactions, there is opportunity to democratize the field to include more common functional groups that engage via non‐classical binding modes. Interrogating excited‐state catalyst conformation, and subsequent interactions with the substrate, will require a multifaceted approach to provide robust induction models and expedite reaction development.

**Figure 10:**
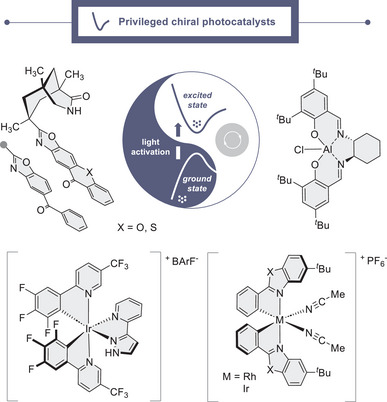
Privileged chiral photocatalysts that have been validated in diverse asymmetric transformations.

An interesting evolutionary observation is that the catalyst blueprints described in this mini‐review (Figure 10)^[^ ^165^ ^]^ have their origins in ground state asymmetric catalysis, where many display remarkable generality and are already considered to be privileged. It logically follows that repurposing ground‐state chiral catalysts that contain well‐defined chromophores will likely lead to new avenues of investigation in asymmetric photocatalysis and possibly mitigate the need for multi‐step synthesis campaigns. It is tempting to speculate that many of the solutions to bridge the reactivity – selectivity divide in asymmetric photocatalysis may have been sitting in laboratory chemical collection for years!

## Conflict of Interests

The authors declare no conflict of interest.

## Data Availability

Data sharing is not applicable to this article, as no new data were created or analysed in this study.
